# Supplementary material for: Protein Carbonylation As a Biomarker of Heavy Metal, Cd and Pb, Damage in Paspalum fasciculatum Willd. ex Flüggé
Source: Plants (Basel). 2019 Nov 16;8(11):513. doi: 10.3390/plants8110513 (PMC6918243; doi:10.3390/plants8110513)
Supplement: Supplementary file 1 [file plants-08-00513-s001.pdf]

Supplementary Material

Protein carbonylation as a biomarker of heavy metal, Cd and Pb, damage in *Paspalum fasciculatum* Willd. ex Flüggé

Manuel Salas-Moreno[a,b], Neyder Contreras-Puentes[b], Erika Rodríguez-Cavallo[b], Jesús Jorrín-Novo[c], José Marrugo-Negrete, Darío Méndez-Cuadro[b*]

[a] Faculty of Naturals Sciences, Biology Department, Biosistematic Research Group, Technological University of Chocó, Quibdó, Colombia

[b] Analytical Chemistry and Biomedicine Group. Exacts and Natural Sciences Faculty. University of Cartagena. Cartagena-Colombia.

[c] Department of Biochemistry and Molecular Biology. University of Cordoba (Spain)

[d] Chemistry Department, Water, Applied and Environmental Chemistry Group, University of Córdoba, Faculty of Basic Sciences, Montería, Colombia

Corresponding authors:

Darío Méndez-Cuadro. **Mailing address**: Analytical Chemistry and Biomedicine Group. Faculty of Exact and Natural Sciences. The Campus of San Pablo, first floor No.109. Cra. 50 #24-120, PA 130015. Cartagena, Colombia, University of Cartagena. **Phone number:** + 57 3015584887. **E-mail**: dmendezc@unicartagena.edu.co

SUPPLEMENTARY TABLES

Table S1. Bioaccumulation (BAF) and Translocation (TF) Factor in *P. fascicdulatum*.

**TFs and BAFs of *Paspalum fasciculatum* Willd**						
**Time Exposure**	**Treatments**					
**Days**	**TC15**		**TC30**		**TC50**	
	TF	BAF	TF	BAF	TF	BAF
**30**	0.12	10.4	0.12	5.10	0.07	5.55
**60**	0.26	ND	0.20	ND	0.18	ND
**90**	0.29	3.87	0.53	4.12	0.12	4.83
**Days**	**TP15**		**TP30**		**TP50**	
	TF	BAF	TF	BAF	TF	BAF
**30**	1.68	0.15	0.80	0.21	0.63	0.74
**60**	0.26	ND	0.12	ND	0.1	ND
**90**	0.27	0.16	0.15	0.31	0.06	0.36

Treatments: TC15 = 15 ppm Cd; TC30 = 30 ppm Cd; TC50 = 50 ppm Cd; TP15 = 15 ppm Pb; TP30 = 30 ppm Pb; TP50 = 50 ppm Pb; ND: Non-determinate

Table S2. Yielding of protein from *Paspalum fasciculatum* leaves and root exposed at Cd and Pb in mining soil

Condition	Time(Days)	Code	Exposed Heavy Metal	µg of protein	µl Extracto protein	Yielding (µg of protein/µl of Extract)
**Control - Roots**	30	R1	Control	146,7	220	0,293
		R2		22,7	220	0,045
		R3		22,7	220	0,045
	60	R1		34,1	220	0,068
		R2		51,5	220	0,103
		R3		277,4	300	0,555
	90	R1		32,3	220	0,064
		R2		34,0	220	0,068
		R3		27,1	220	0,054
**Control - Leaves**	30	R1	Control	101,3	220	0,202
		R2		139,7	220	0,279
		R3		146,7	220	0,293
	60	R1		127,2	200	0,424
		R2		127,7	200	0,426
		R3		129,2	200	0,431
	90	R1		269,0	300	0,538
		R2		464,3	300	0,929
		R3		466,7	300	0,933
**TC30-Leaves**	30	R1	30ppm Cd	96,0	200	0,192
		R2		72,0	150	0,144
		R3		78,5	200	0,157
	60	R1		71,8	200	0,143
		R2		86,1	180	0,172
		R3		95,7	150	0,191
	90	R1		122,8	200	0,246
		R2		110,5	180	0,221
		R3		73,7	120	0,147
**TC30-Root**	30	R1	30ppm Cd	21,8	220	0,043
		R2		9,6	220	0,019
		R3		16,7	300	0,033
	60	R1		61,9	300	0,124
		R2		44,0	300	0,089
		R3		45,2	300	0,090
	90	R1		30,6	220	0,061
		R2		32,3	220	0,064
		R3		34,9	220	0,069
**TP50-Leaves**	30	R1	50ppm Pb	168,5	220	0,336
		R2		240,1	220	0,480
		R3		213,0	220	0,426
	60	R1		140,7	200	0,469
		R2		126,3	200	0,421
		R3		143,7	200	0,479
	90	R1		242,8	200	0,485
		R2		283,3	200	0,567
		R3		273,8	200	0,547

Table S3. Relation values C.I of exposition to *Paspalum fasciculatum* by Cd and Pb with control.

Days	C.I. Exposed to metal/C.I. control		
	30 ppm Cd/Control		50 ppm Pb/Control
	Roots	Leaves	Leaves
**30**	4,7	9,9	ND
**60**	3,2	2,3	2,8
**90**	2,0	ND	2,1

C.I.: Carbonyl Index.

Supplementary Figures

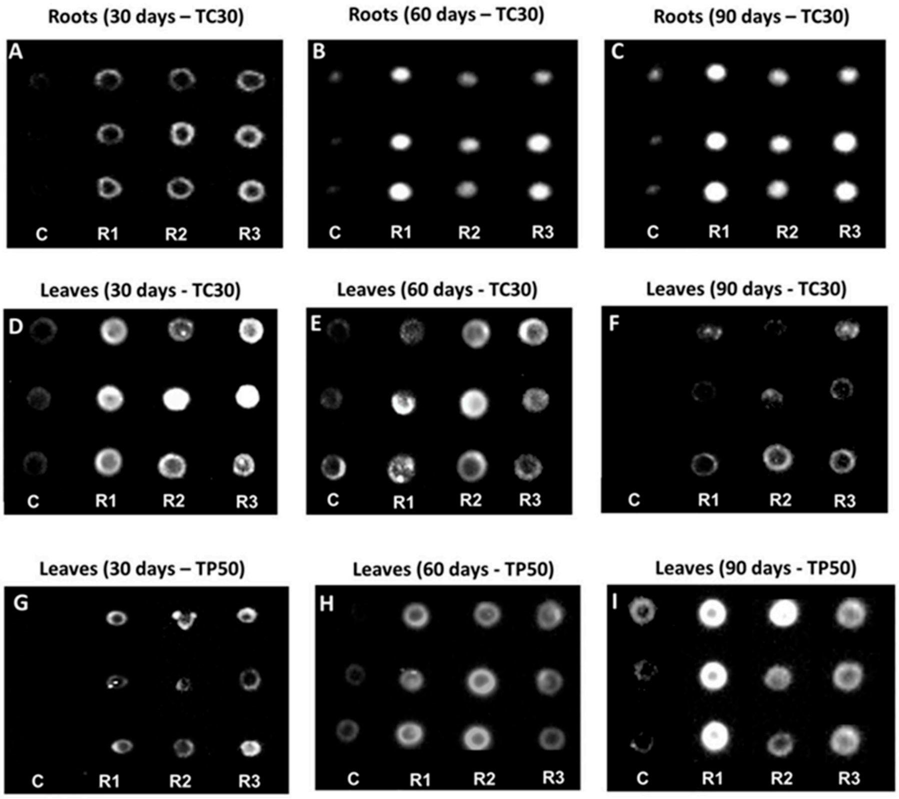

SF 1. Oxidative dot-blots of roots and leaves proteins from *Paspalum fasciculatum* Willd (ex Flüggé) (Poaceae). Panels show chemiluminscense signal of carbonylated proteins for control (C) and exposed (R) samples. Panels **A-C:** correspond to roots exposed to 30 mg kg^{-1} Cd for 30, 60 and 90 days, respectively. Panels **D-C:** leaves exposed to 30 mg kg^{-1} Cd for 30, 60 and 90 days, respectively. Panels **G-I:** leaves exposed to 50 mg kg^{-1} Pb for 30, 60 and 90 days, respectively.
